# A hypothesis on a role of oxytocin in the social mechanisms of speech and vocal learning

**DOI:** 10.1098/rspb.2017.0988

**Published:** 2017-08-23

**Authors:** Constantina Theofanopoulou, Cedric Boeckx, Erich D. Jarvis

**Affiliations:** 1 Section of General Linguistics, University of Barcelona, Barcelona, Spain; 2 ICREA, Passeig Lluís Companys 23, 08010 Barcelona, Spain; 3 Universitat de Barcelona Institute for Complex Systems, Barcelona, Spain; 4 Department of Neurogenetics of Language, Rockefeller University, New York, NY, USA; 5 Howard Hughes Medical Institute, Chevy Chase, MD 20815, USA

**Keywords:** oxytocin, language, vocal learning, birdsong, dopamine

## Abstract

Language acquisition in humans and song learning in songbirds naturally happen as a social learning experience, providing an excellent opportunity to reveal social motivation and reward mechanisms that boost sensorimotor learning. Our knowledge about the molecules and circuits that control these social mechanisms for vocal learning and language is limited. Here we propose a hypothesis of a role for oxytocin (OT) in the social motivation and evolution of vocal learning and language. Building upon existing evidence, we suggest specific neural pathways and mechanisms through which OT might modulate vocal learning circuits in specific developmental stages.

## 1. Vocal learning (speech and song) and social experience requirements

Vocal learning is the ability to imitate sounds, found to date in only a few independently evolved species of mammals (humans, bats, cetaceans, sea lions and elephants) and birds (songbirds, parrots and hummingbirds) [1,2]. It is distinct from both auditory and vocal usage learning, which are more ubiquitous among species, and are necessary but not sufficient for vocal learning [3,4].

Early language acquisition is very strongly shaped by social interactions [5]. These social interactions include social motivation for speech learning, emphasized since the dawn of developmental psychology [6]. More recently, social motivation for speech learning has been viewed as a type of social learning [5,7]. Even other forms of sensory–motor learning can involve social feedback [8], and plausibly speech learning could be using a similar mechanism. Several laboratories have experimentally begun to test this hypothesis in humans, and determine to what extent social interactions that modulate attentional, sensory and sensorimotor mechanisms promote language learning. For example, phonological features of babbling are shaped developmentally by social feedback [9] and child speech-related vocalizations (non-cry, non-laugh and non-vegetative) are more likely to receive adults' responses, and in turn, a child's vocalization tends to be speech-related, if the previous speech-related vocalization received an immediate adult feedback [10]. That is, babbling both regulates and is regulated by social interactions, where an infant is socially motivated to learn how to speak, because this learning process is socially rewarding.

This hypothesis, though, needs to be tested with experimental manipulations in non-human animals. The few examples we have from children reared under conditions of social isolation can just partially inform us on the importance of social feedback in language acquisition, both in the auditory and speech domains (as in the famous case of Genie [11]). Kuhl *et al.* [12] managed to tease apart interpersonal interactions from sensory information, by exposing infants to either audiovisual stimuli or just audio recordings, showing that successful language learning is impossible without social cues. Nonetheless, this experiment was made for second-language learning, leaving unanswered the question of whether the primary speech learning mechanisms can be dissociated from the relevant motivational and rewarding mechanisms provided by social interactions. Further, these experiments tested mainly auditory learning/perception, which is thought to have a different mechanism and brain pathway than speech production learning [4].

Building on the extensive comparative literature on the role of motivation and social decision making in sensory–motor networks [13,14], we think that animal models could shed more light on this issue, and specifically, vocal learning species. Of the non-human vocal learners, songbirds have been studied the most. Songbirds' vocal-learning ability displays parallels to human speech learning, having undergone convergent evolution, at the level of behaviour, neural connectivity and gene expression specializations in song and speech brain regions [2,15,16]. Thus, by dissecting the social mechanisms of vocal learning in songbirds, we could illuminate how social interactions shape vocal learning in humans.

Like human infants, juvenile songbirds learn their songs from social tutors. In laboratory tests, juvenile zebra finches learn best from a live tutor [17]; learning from purely tape recorded songs is less effective, and for some species, often not effective at all [18]. This strong social requirement makes the zebra finch a good candidate for modelling the impact social factors have on human vocal learning. Under conditions between live tutor versus speakers producing song, there are intermediate levels of vocal learning. For example, blindfolded zebra finches interacting with their tutors via grooming or pecking do learn some song, probably in a similar way that blind humans acquire a fully fledged language [17]. Tchernichovski *et al.* [19] have been able to get young zebra finches motivated to learn how to sing without a live tutor, by having them perform an operant conditioning task for the song playback from a fake bird model. When the juveniles have to peck on a key to induce song playbacks from the model, they eagerly keep pecking, and within days to weeks begin to start copying the song from the model [20]. However, if the key is not present and song is played from the model only or the speaker is removed from the model with song played in another location, the juveniles learn very little if at all [21]. These findings indicate that live tutors or fake model birds emit more robust singing social stimuli giving rise to enhanced vocal development, compared to when juvenile songbirds are reared with speakers. This suggests that there could possibly be a social reward mechanism enhancing sensorimotor imitation, a hypothesis that remains to be tested, particularly at the neural and molecular level.

Even though this live versus tape-tutor paradigm could have functioned as the best springboard to study the social mechanisms of vocal learning, researchers have mostly used it to control the auditory parameters the birds get exposed to (e.g. [21]). In addition, since the discovery that male zebra finches alter the structure of their song, gene expression and physiology in song nuclei depending on whether they sing to no one in particular (undirected singing) or to attract a female (directed-singing) [22,23], many studies have focused on adult social interactions after vocal learning is complete. There is a paucity of studies dealing with how social interactions mechanistically affect vocal learning in juvenile songbirds. Among these, Chen *et al.* [24] show that social influences on attention to song enhance vocal learning: tutors altered the structure of their song when directing it to juveniles, reminiscent of the special ‘motherese’ way humans speak when addressing their speech to infants.

Deciphering the mechanisms of the social motivation of vocal learning, and determining whether the mechanism of social motivation to learn vocalizations can be dissociated from the act of vocal learning, we believe requires figuring out the circuit and molecular mechanisms. Towards this end, we propose that the neuropeptide oxytocin (OT) and its social reward circuitry make a very good candidate that could control the social reward mechanisms for vocal learning.

## 2. Oxytocin as a good candidate to control social motivation of vocal learning

Oxytocin, depending on the brain region and release site, acts as a hormone, neuromodulator or neurotransmitter that functions through its receptor (OTR) to regulate a diverse set of biological processes: pregnancy and uterine contractions, milk ejection, attachment between mothers and their young, bond formation, copulation and orgasm, suppression of stress, thermoregulation, olfactory processing, eye contact and recognition of familiar individuals [25], with the caveat that some functions are specific to one lineage, such as mammals. OT is thought to have its effect on many systems because it is most prominently expressed in hypothalamic OT neurons that project to many brain regions where the receptor is located [26,27]. Recent studies attest that OT enhances socially reinforced learning in humans and rhesus macaques [28,29], while other studies show its involvement in vocal and auditory behaviours (see references herein). As a result, Theofanopoulou [30] put forth the hypothesis that OT might be implicated in cognitive aspects of language processing in humans. Here we adduce more evidence also for a role in the social motivation of language learning. We further sketch out possible mechanisms for social motivation for vocal learning in vocal-learning species. With regard to gene terminology, we have adopted a universal nomenclature based on sequence identity and gene synteny, using the same gene name OT and OTR across vertebrates [31].

### (a) Vocal non-learners

We first note that OT appears to have a role in auditory–vocal communication even before vocal learning evolved, as such a role can be found in vocal non-learning species that span the vertebrate phylogeny, from fish to mammals. Goodson & Bass [32] found that OT in midshipman fish modulates the burst duration in the innate vocalizations that sneaker males and females produce in non-reproductive contexts. OT immunoreactive cell groups are distributed throughout their vocal–acoustic circuit, from the midbrain to the forebrain [33]. In rats, OT enhances both inhibitory and excitatory synaptic currents in the hypoglossal motor nucleus which innervates the tongue muscles, thus potentially controlling rat vocalizations [34]. In mice, Winslow *et al.* [35] found that infant OT-KO (knock-out) animals were less vocal than wild-type (WT) controls during separations from the mother and peers. Likewise, Takayanagi *et al.* [36] observed fewer ultrasonic vocalizations emitted by infant OTR-KO compared with wild-type mice in a social isolation paradigm. Marlin *et al.* [37] demonstrated that when inexperienced virgin females are given OT intraperitoneally or through optogenetic stimulation of hypothalamic OT neuronal axons that project into the auditory cortex, and then co-housed with a mother and her litter, their retrieval of vocalizing pups was effective as if they were the mother. Follow-up studies showed that OTR levels are remarkably lateralized with higher expression in neurons of the left auditory cortex [26]. These observations in vocal non-learners strike us as particularly relevant, as auditory and vocal learning/language circuits in humans are mainly left-lateralized [38], and are either left- or right-lateralized among different species of song-learning birds [39].

Based on these findings, we suggest that OT has a role in social motivation of auditory and vocal communication behaviours in vocal non-learners, and that a lateralized function in the auditory cortex may have been present before vocal learning and language evolved. Even though less likely, it could still be possible that OT influences the social motivation for vocal learning through the OTR receptors in innate brainstem circuits, including two auditory brainstem nuclei, the nucleus magnocellularis (NM) and the nucleus laminaris (NL) and in the vocal motor neurons (nXIIts) in songbirds [40].

### (b) Vocal learners

In humans, intranasal OT administration modulates semantic integration in speech comprehension [41]. In autistic patients, the oxytocinergic system has been repeatedly indicated to function aberrantly. Specifically, Rijlaarsdam *et al.* [42] identified a significant OXTR rs53576 genotype by OXTR methylation interaction associated with communication problems in autistic patients, while Zhang *et al.* [43] found that autistic children with higher plasma OT concentrations tended to have less impairment of verbal communication. In turn, after OT intranasal administration, autistic patients had a more efficient and long-lasting performance in a speech comprehension task [44,45]. Based on findings that intranasal administration of OT crosses the blood–brain barrier and binds to areas where the receptors are located [46], we can interpret these studies as bearing directly on our hypothesis.

In songbirds, experimental manipulation of the oxytocinergic system with OT agonist and antagonist have been made mostly in the context of pair-bonding and aggression, with very few and some controversial reports on how these treatments affected singing, probably due to different treatment sites [47–49]. Nevertheless, OT has been found to affect the amount of directed singing to females [48]. These findings in vocal learning species indicate that OT may also have a social enhancement for aspects of auditory processing and learned vocal communication.

### (c) Neural pathways

**Figure 1.**
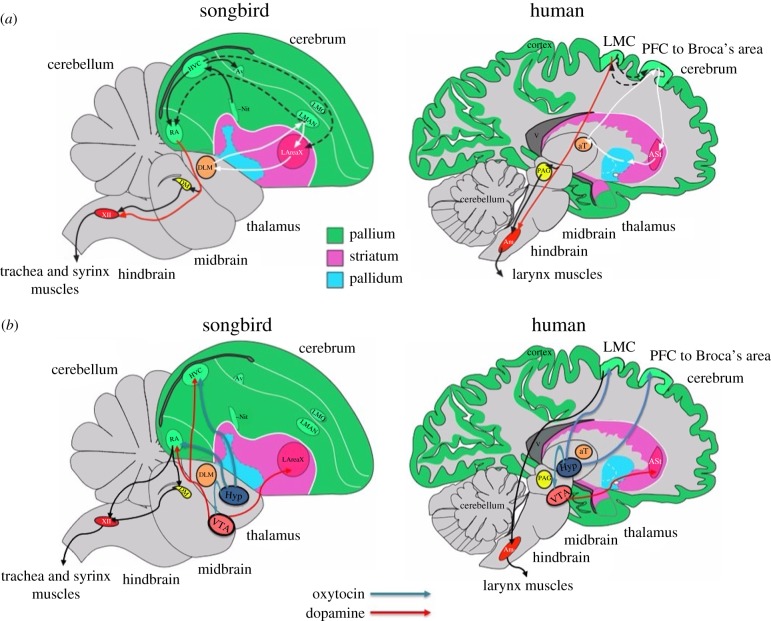
Summary diagrams of vocal learning systems in songbirds and humans. (*a*) Vocal learning circuits. Red arrows, the direct posterior forebrain projection to vocal motor neurons in the brainstem. White lines, anterior forebrain circuit. Dashed lines, connections between the anterior and posterior vocal motor circuits. (*b*) Proposed oxytocinergic and dopaminergic projections into the vocal learning circuits. In songbirds, we propose oxytocinergic neurons from the Hyp project to the RA, HVC and VTA; VTA makes a strong dopaminergic projection to LAreaX and weaker ones to HVC and RA. In humans, we propose oxytocinergic neurons from the Hyp project to the LMC, Broca's area and the VTA; VTA makes dopaminergic projections to the ASt. Black arrows, connectivity of the proposed system with the brainstem. Abbreviations: HVC, HVC nucleus; LMAN, lateral magnocellular nucleus of anterior nidopallium; RA, robust nucleus of arcopallium; Area X, area X of the striatum; Hyp, hypothalamus; VTA, ventral tegmental area; DLM, dorsal lateral nucleus of the medial thalamus; Av, nucleus avalanche; LMO, lateral oval nucleus of the mesopallium; NIf, interfacial nucleus of the nidopallium; DM, dorsal medial nucleus of the midbrain; XII, 12th nucleus, tracheosyringeal part; PFC, prefrontal cortex; LMC, laryngeal motor cortex; A St, anterior striatum; PAG -periaqueductal grey; aT, anterior thalamus; Am, nucleus ambiguus of the brainstem. Note: The position of Broca's area is shown here more medially for simplicity. (Adapted from [4,16].)

In order for our hypothesis to have some validity, OT would be expected to innervate vocal learning circuits directly, that in turn would express the OTR, or indirectly via other motivation/reward circuits that, in turn, innervate vocal learning circuits [50]. All vocal learning species examined to date (humans and the song learning birds) have a highly specialized forebrain circuit that controls learning and production of learned sounds (figure 1*a*) [4,16]. Best studied in songbirds, the pathway consists of an anterior forebrain circuit that controls vocal imitation and a posterior circuit that controls production of learned vocalizations. The anterior forebrain circuit consists of LMAN in the cortical region, Area X in the striatum and aDLM in the thalamus, which form a pallial-basal ganglia-thalamo-pallial loop (figure 1*a*). When Area X is lesioned in juveniles, the birds are not able to crystallize onto a learned song, as their vocalizations remain variable. Conversely, when LMAN is lesioned, the bird instantly crystallizes onto what it had learned up to that moment [51]. These and other findings lead to one interpretation being that during the juvenile vocal learning period, Area X injects stereotypy, whereas LMAN injects variability into the vocalizations, and the two opposing functions enable vocal imitation [2,51]. After learning is complete, lesions in adults, such as in Area X, lead to deficits in song sequencing (or production) similar to stuttering in humans [52,53]. The posterior pathway in songbirds consists of the HVC and RA, thought to control sequencing and acoustic structure of syllables, respectively. In humans, the analogous anterior pathway has been proposed to be a cortical-basal ganglia-thalamo-pallial loop involving Broca's area (LMAN analogue), part of the anterior striatum (ASt) and the anterior thalamus; the analogous human posterior pathway has been proposed to include the laryngeal motor cortex (LMC; figure 1*a*), with different cortical layers representing songbird HVC (layer 3) and RA (layer 5) [2,16]. This forebrain vocal pathway is either absent or limited at best in vocal non-learning species, including non-human primates and mice [54,55]. But all vocal learning and non-learning species have a more comparable auditory forebrain pathway, involved in auditory learning, as described above for the mouse pup retrieval experiments.

The OTR is expressed broadly across cortical and subcortical brain regions, in both mammals and birds, including humans [27,56,57]. However, in different species there are brain regions with enriched OTR expression relative to all other brain regions, and they often correlate with differences in social behaviours between species [58]. We are not aware of anyone determining if there is enriched specialized expression (increased or decreased relative to adjacent brain regions) in speech brain regions in humans. In songbirds, some limited expression analyses in the posterior pathway revealed differences between species, with specialized upregulation of OTR in HVC and downregulation in the RA compared with the surrounding motor regions in zebra finches, that sing one simple song and higher expression (although not shown) in white throated sparrows, a species that sings at least two different songs [40]. One prediction from these findings would be that human LMC layer 5 neurons may have downregulation of OTR relative to layer 5 neurons of the adjacent non-speech cortex. We noted from our examination of fig. 1 of [58] that there is layer-specific expression of OTR in the motor cortex that is different across rodent species. We also predict some within-species differences, such as in songbirds where females lost the vocal learning trait [59] and would not be expected to have forebrain vocal OT neuron innervation.

In terms of possible indirect interactions through other reward brain circuits, hypothalamic OT neurons innervate the ventral tegmental area (VTA), which innervates the vocal learning systems in humans and songbirds [60,61]. The VTA releases dopamine (DA) mainly to striatal brain regions and some cortical brain regions, including vocal learning regions in songbirds [62]; through its DA receptors it is thought to reinforce learning and motivated behaviour. There is a plethora of evidence in the mammalian literature showing that OT neurons in the hypothalamic paraventricular and supraoptic nuclei (PVN and SON) send projections to the VTA, and stimulate DA neurons there [63–66]. Consistent with this, in the last several decades, a number of studies have shown OT–DA interactions in many social behaviours [67,68]. The VTA expresses OTR [69,70] and injection of OT into the VTA of rats increases DA release, inducing penile erection [63,71,72]. Intracerebroventricular injection of an OTR antagonist attenuates DA agonist-stimulated DA release and the pro-erectile effect [64]. Peris *et al.* [73] infected mice with a Cre-inducible adeno-associated virus that drives the expression of an OTR-fluorescent reporter in the VTA and found that OTR-expressing neurons in VTA project to the nucleus accumbens, prefrontal cortex, the extended amygdala and other forebrain regions; also some of these neurons were identified as DA neurons. Bromberg-Martin *et al.* [74] have also shown that DA neurons within the VTA encode motivationally salient signals. Thus, OT, by modulating activity within the DA system, may alter the assignment of motivational salience.

Lastly, since OT can bind to one of the vasopressin/vasotocin receptors (vasopressin/vasotocin receptor 1A; AVPR1A or V1AR) with equal affinity as it does to the OTR [75], we do not exclude the possibility that OT may be playing a role in the social motivation for vocal learning via this receptor too. It is also the only vasopressin/vasotocin receptor thus far found to be expressed in vocal learning regions [40] and to be involved in singing behaviour [76].

Taking the behavioural, circuit and molecular findings together, we suggest that OT and the circuits it functions in are good candidates for the long-hypothesized motivation and reward mechanism of vocal learning. Part of the mechanisms may have been present before vocal learning evolved, but part of it may be specialized in vocal learning circuits and behaviour. With this information, we propose a testable mechanism, either via direct influence on vocal learning pathways or indirect through the VTA DA neuron pathway.

## 3. Proposed neural and molecular mechanisms

In this section, we consider the *when, where* and *how* OT might modulate socially motivated vocal learning behaviour.

For the *when*, we consider the three major stages vocal learners are known to acquire their ability to imitate vocalizations: sensory, sensorimotor practice and crystallization. In some species these stages can be distinctly separate, and in others they overlap. In the first sensory infant/nestling phase, vocal learning animals and humans acquire auditory memories of the vocalizations that they hear through social interactions [15]. In this phase, it is not necessary that the animal or child imitate or even vocalize. In the second, sensorimotor phase, as in other cases of sensorimotor learning, vocal learning proceeds through a reinforcement learning mechanism [50,61], where juvenile song-learning birds begin to produce semi-imitated vocalizations, and evaluate their own motor output via sensory feedback and reinforce it only if it closely matched the predicted outcome [77]. A mechanism proposed for reinforcement is the variability observed in juvenile song, suggestive of a motor exploration [78], with reinforcement (or error) neural signals guiding song imitation [79]. Likewise, human infants in this phase appear to experiment with uttering articulate sounds, but without yet producing recognizable words (i.e. babbling). Again reward behaviours, e.g. when parents complement their child with excited words, clapping, smiles and hugs, after their first speech-related attempts, reinforces speech learning. In the third crystallization phase, as they become adults (i.e. puberty phase in humans), song-learning birds and speech-learning humans complete the development of their vocal repertoire, and the ability to learn new vocalizations/languages is either shut down (e.g. zebra finches) or made more difficult (e.g. canaries and humans). However, if a song-learning bird is removed from its conspecifics before this phase is complete, it will take significantly longer for the animal to crystallize on a repertoire [80]. We propose that OT will have its effects during the sensory and sensorimotor phases of vocal learning, and less so during or post crystallization, because the first two phases are more dependent on social experience. It is also likely that the same mechanisms could apply throughout life, but at a more reduced level.

For the *where* and *how*, during the sensory phase, we propose that OT could enhance the formation of socially driven auditory memories that impinge on the vocal learning circuit. This could occur by a direct projection of OT hypothalamic neurons into the auditory cortex, as seen in adult female mice for pup retrieval, or by direct projections to the vocal learning pathway brain regions. For the former possibility, auditory input into juvenile HVC from a playback has been found to modulate its neural connectivity and function in song production [81]. We propose that when a vocal learning infant/juvenile hears vocalizations generated from a conspecific, there could be an associated increase in OT release into the auditory and/or specialized vocal learning brain regions to strengthen the newly formed synaptic interactions to hold onto the memories and shape the vocal learning pathway. Similar to DA circuits (see below), this strengthening and shaping could occur by OT binding to OTR in neurons of the auditory and vocal pathways that receive excitatory and inhibitory inputs for the auditory–vocal memories. A prediction of this hypothesis is that if the auditory signals are not from a social individual, the auditory processing circuits would still process the sounds and form auditory memories of them, but the OT circuit would not strengthen the auditory input to the vocal learning circuit for eventual imitation of the sounds.

During the sensorimotor learning phase, we propose again that OT input to the auditory and vocal learning pathways could be activated, but this time by positive social feedback (auditorily or by other means) from conspecifics when the juvenile produces more accurate copies of the learned vocalizations. The positive feedback could help strengthen the connections that control production of the more accurate copy of the vocalizations. But could OT also modulate imitation of vocalizations during sensorimotor practice independent of immediate social input from others? Although this would move us away from a direct social role of OT in vocal learning, we consider the possibility that self-motivation and even purely vocal learning mechanisms independent of immediate social mechanisms could also be involved. For this possibility, we turn to studies on DA.

As described earlier, there is a robust VTA DA-neuron projection to vocal learning nucleus Area X (figure 1*b*) [60,62]. An analogous vocal learning region has been found in the human striatum, with many of same gene expression specializations as in songbird Area X [16,82]. VTA also makes a weaker, but still relatively prominent, projection to the vocal production nuclei HVC and RA, and receives input from an auditory area around RA necessary for vocal learning [83]. DA levels in Area X are higher during directed singing (to females) than undirected singing, due to differential activity of the re-uptake transporter (a noradrenaline transporter in birds), in the VTA axons within Area X [84]. When this transporter is pharmacologically blocked, DA levels during undirected singing reach the levels of DA release during directed singing [84]. Unilateral lesions of the VTA dopaminergic projections reduce singing-driven Immediate Early Gene (IEG) expression in Area X in both contexts [60]. More recently, Gadagkar *et al.* [79] showed that the VTA DA neurons that project to Area X encode performance error-and-reward during singing, where these neurons are suppressed when the bird simultaneously hears distorted feedback syllables and are activated when they hear undistorted syllables. It is plausible to hypothesize that such *performance* signals might subserve vocal *learning* in juvenile animals, when the songbird monitors if the vocal output produced matched ‘the desired tutor outcome, and also the predicted probability of achieving the desired outcome’ [79]. Recently, Chen *et al.* [24] found that in juvenile animals the percentage of DA neurons expressing EGR-1 (an IEG) in the VTA was significantly higher in socially tutored juveniles relative to passively tutored juveniles with playbacks of songs from a speaker or untutored juveniles, indicating that this neural correlate might be responsible for the differences in vocal-learning performance.

We propose that OT might have a role in both the social motivation and the sensorimotor mechanisms of vocal learning via hypothalamic OT action on VTA DA-neurons that project to Area X and other song nuclei (figure 1*b*). During sensorimotor practice, there could be self-induced motivation of the OT → VTA → song nuclei circuits to help strengthen connections within the circuit when the tutee's produced song matches his auditory memory of the tutor's song. After vocal learning is complete, the presumed downregulation of OTR in several vocal learning nuclei (relative to the surrounding brain regions) in zebra finches may contribute to crystallization and shutting off the ability to further imitate from conspecifics. For the latter part of the hypothesis to be plausible, one would need to determine if there are higher levels of OTR in these song nuclei during juvenile development.

It is important to mention that up to now the only well-studied hypothesis of where the VTA gets its input for vocal learning functions has been articulated by Riters [85] and colleagues based on their studies on European starlings. According to them, it is the projection from the medial preoptic nucleus (mPOA) to the VTA that is crucial for social reward-related functions of vocal learning. Our hypothesis shifts the focus from the mPOA to the hypothalamus, because of the OTR expression in song-learning nuclei and suggestive direct influence of hypothalamic OT on singing. The two hypotheses could be complementary: OTRs are also expressed in the mPOA and mPOA neurons (at least in mammals) that project to the VTA (or to the PVN and from there to VTA) [86] play a role in social bonding regulation and maintenance [72]. That is, the OT input to the VTA could be originating both from the hypothalamus (PVN/SON → VTA) and the mPOA (mPOA → VTA or mPOA → PVN → VTA).

In humans, building on OTR expression patterns in the brain [27,56,57], we propose that OT neurons might project directly from the hypothalamus to the LMC and Broca's area or indirectly to them and other speech-regions through the VTA (figure 1*b*). Regarding the latter, there is evidence that OT administration enhances activation in the VTA of humans [87]. In this manner, OT might affect VTA's DA output to the anterior striatum speech region [88] and LMC [54,61], and from there (LMC) to the vocal motor nucleus ambiguous of the brainstem. Given these similar findings in humans, we see no reason to propose a fundamentally different mechanism for the sensory and sensorimotor learning phases of vocal learning in humans or other vocal learning species.

An alternative route through which OT could also affect the social motivation for vocal learning is through its hormonal action via the hypothalamic–pituitary–adrenal axis, known for attenuating the stress response [89] and thus making social learning more efficient. However, we deem this possibility as less likely, given that the OTR is found in the auditory cortex and in speech/song areas, most likely directly affecting vocal learning.

## 4. Proposed experiments to test hypothesis

In this final section, we offer some proposed experiments that would validate or falsify some of the key tenants of our hypothesis.

A prediction of our hypothesis that OT controls the social motivation to imitate vocalizations in vocal learners, is that blocking OT in the brain, and more specifically its targets to the OTR in auditory cortex, vocal learning neurons and/or VTA-DA neurons during the sensory and sensorimotor phases would prevent vocal learning from live social or model tutors. Conversely, activating OT in these circuits, when a young juvenile hears novel vocalizations from a live tutor or a tape recorder, would potentially cause the juvenile to imitate the song heard better and also treat that tape recorder as more of a social object. This would also mean that OT neuron activation and release, and activation of OTR in the target brain regions, would also change in the same direction. It is not feasible or ethical to conduct such experiments in humans, but they can be conducted in a non-human vocal learning species, such as songbirds.

Because our hypothesis is at its infant stage, informing and testing the hypothesis further will also require a great amount of more descriptive research. This includes: (i) a detailed expression analyses of OT, OTR and associated family of genes (vasopressin/vasotocin and its receptors) in the vocal communication brain regions throughout development and adulthood, across multiple vocal learning and non-learning species; (ii) analyses of coding sequence and regulatory regions of these genes to determine if there are convergent genetic changes in vocal learning species that could explain brain functional or expression differences, respectively and (iii) physiology analyses of OT neurons and OT release during vocal learning and language acquisition. Some of these more descriptive experiments can be done with humans and non-human primates, and thus offer a more direct window to inform our hypothesis on OT function in language.

## 5. Conclusion

We have sketched out what we consider a plausible hypothesis of a role for OT in the social motivation of vocal learning and language. This hypothesis, if validated, would fill in a gap in our knowledge of the main molecule(s) that control the social motivation for vocal learning. With this hypothesis, we are able to assemble disparate pieces of knowledge into a greater whole, with OT as a nexus. As in all hypotheses, there are parts that have weaknesses in ours, such as whether OT modulation of vocal learning circuits and thus language are direct or indirect. For these, we propose plausible alternative mechanisms that can be tested and modified with new knowledge. Overall, though, we find it hard to come up with a better viable alternative hypothesis, given the current state of knowledge. Thus, we believe the hypothesis we propose at this time is the most attractive one worth testing.

## Data accessibility

This article has no additional data.
